# Water-Ionic Liquid Binary Mixture Tailored Resorcinol-Formaldehyde Carbon Aerogels without Added Catalyst

**DOI:** 10.3390/ma12244208

**Published:** 2019-12-14

**Authors:** Balázs Nagy, István Bakos, Erik Geissler, Krisztina László

**Affiliations:** 1 Department of Physical Chemistry and Materials Science, Budapest University of Technology and Economics, H-1521 Budapest, Hungary; nagy.b555@gmail.com; 2 Institute of Materials and Environmental Chemistry, Research Centre for Natural Sciences, Eötvös Loránd Research Network, H-1519 Budapest, Hungary; bakos.istvan@ttk.mta.hu; 3 Laboratoire Interdisciplinaire de Physique, Université Grenoble Alpes and CNRS, 38402 Grenoble Cedex, France; e.geissler@orange.fr

**Keywords:** RTIL, sol-gel reaction, pore size distribution, nitrogen adsorption isotherms, SAXS, SEM

## Abstract

The potential applications of mesoporous carbon aerogels are wide-ranging. These gels are often obtained from resorcinol-formaldehyde (RF) hydrogel precursors. The sol-gel method in this synthesis provides an efficient and versatile means of product control through systematic variation of process conditions, such as pH, stoichiometry, concentration, catalyst, further additives, etc., in addition to the drying and pyrolytic conditions. Here, a novel means of tuning the texture of carbon aerogels is proposed. Water-1-ethyl-3-methylimidazolium ethyl sulfate ([emim][EtSO_4_] mixtures constitutes a polycondensation medium that requires no added catalyst, thus yielding an intrinsically metal-free carbon aerogel after pyrolysis. We also show that the carbon morphology is tailored by the supramolecular structure of the aqueous ionic liquid. The results of scanning electron micrographs, low-temperature nitrogen adsorption/desorption isotherms, and small-angle X-ray scattering (SAXS) confirm that changing the initial water concentration from 9 to 55 wt % gives rise to systematic alteration of the mesopore size and volume, as well as of the bead size. The pore structure becomes consolidated only when the water content exceeds 25 wt %. When the water content reaches 55 wt %, the bead size increases by two orders of magnitude. The electrocatalytic performance, however, is compromised, most probably by structural defects.

## 1. Introduction

The ability to produce carbon gels with tailored 3D shape and pore morphology is a direct result of the flexibility of the most frequently employed sol-gel synthesis method. Moreover, an extensive set of routes exists by which the surface chemical properties of these nanostructured carbon materials can be modified, either simultaneously or independently.

Carbon aerogel (CA) precursors are generally obtained by catalytic polycondensation reactions between synthetic or natural (poly)hydroxy benzenes and formaldehyde, most often in aqueous medium [1,2,3]. Tuning the fundamental properties of the carbon precursor hydrogels is achieved by adjusting the reaction parameters: molar ratio of the reagents, pH, choice of catalyst, inclusion of further additives, overall concentrations, temperature trajectory, and duration of aging [2,3,4,5,6]. Each of these factors, either individually or in combination, influences the structure of the hydrogel. A further significant variation arises in the choice of the solvent removal technique [7,8]. The final form of the carbon aerogels is obtained by pyrolysis, sometimes followed by activation. 

Numerous possibilities exist for creatively modifying the general route of the polycondensation reaction, which is most frequently performed in water. Already in the solution/sol stage, the addition of co-monomer(s), salt, nanoparticles, the choice of catalysts, etc. influences the polymerization reaction and the morphology of the hydrogel. The method of solvent removal, or other subsequent treatment, is also a valuable tool for materials scientists. 

In this paper, we consider an additional parameter, the chemical nature of the reaction medium. Although originally developed potentially for use as electrolytes in batteries, a broad variety of room-temperature ionic liquids (RTILs) has been employed for carbon aerogels in several ways [9]. While an excellent overview of the progress in the field of organic and carbon gels has recently been published by Arenillas et al. [3], the potential of ionic liquids in their synthesis remains generally unrecognized.

RTILs, a family of salts that is liquid at ambient temperature, consist of bulky, asymmetric organic cations paired with a wide range of smaller, usually inorganic, anions. They may themselves be CA precursors. Carbonization of polymerizable, high N-content metal-free RTILs yields micro-mesoporous carbon materials. The size of the anion and the alkyl chain decorating the cation influences the pore size distribution, which in turn affects both the kinetics and the activity of the catalytic performance. Owing to the 2%–5% N/C ratio, where the nitrogen is mainly in quaternary and pyridinic form, the oxygen reduction reaction (ORR) activity is found to be comparable to Pt/C [10]. N-doped carbon aerogels have been obtained respectively from 1-butyl-3-methyl-pyridinium dicyanamide and 1-ethyl-3-methyl-imidazolium dicyanamide RTIL precursors, with salt templating in which an inorganic eutectic salt mixture of various alkali metal and zinc chlorides was used in completely water-free conditions. Dual-doped carbons containing nitrogen and boron have also been synthesized in a similar way from 1-ethyl-3-methyl-imidazolium tetracyanoborate [11]. The use of RTILs as a novel medium is an expanding field of interest. Despite their high price, they are one of the most promising solvents/reaction media of the future, considering their numerous advantages (non-flammability, low vapor pressure, high-temperature stability, reusability, etc.). It has been demonstrated that RTILs can be used advantageously as a medium for ionothermal conversion of biomass. In 1-butyl-3-methylimidazolium tetrachloroferrate, carbon materials with unique morphological properties have been obtained from various carbohydrates, although the surface area of the resulting products was relatively low (50–155 m^2^/g). Owing to the very low vapor pressure of the ionic liquid (IL), the carbon synthesis can take place practically at ambient pressure. Furthermore, the choice of the anion can also lead to carbon matrices possessing magnetic properties [12].

When resorcinol-formaldehyde (RF) gels are synthesized in an RTIL, the ionic liquid does not necessarily function only as a simple reaction medium but may act concurrently as a catalyst and/or template. Yang et al. [13] investigated a set of resorcinol-formaldehyde carbon aerogels synthesized in aqueous 1-alkyl-3-methylimidazolium-based ionic liquids, and the vacuum dried sample was pyrolyzed at 800 °C in nitrogen flow. The IL served merely as a template, but traces of nitrogen and boron from the ionic liquid were detected in the carbon aerogel by X-ray photoelectron spectroscopy (XPS). The alkyl chain length, the anion, and the IL/water ratio were recognized as factors that influence the micro-mesoporous characteristics of the carbon aerogel, although no systematic trend was observed in the textural parameters. Most of the low-temperature nitrogen adsorption isotherms exhibited low-pressure hysteresis, a sign of poorly developed pore structure. The surface area varied between 380 and 590 m^2^/g, compared to the 370 m^2^/g of the IL-free condition. Another group used hexadecyl-2,2-dimethyimidazolium tetrahydroborate as a template in IL concentrations of at most 7 wt %. After removal of the water at 100 °C, additional heat treatment at 830 °C in water vapor-nitrogen flow yielded a porous carbon with a surface area of 400 m^2^/g. The addition of iron salt to the template doubled the surface area of the sample, which also showed excellent electrochemical performance [14]. Other authors have reported that normal 1-alkyl-3-methylimidazolium bromide RTIL in the resorcinol-formaldehyde precursor solution can play three roles simultaneously, that of a soft template, a source of nitrogen, and, partially, of carbon. Using RTIL led to a porous carbon with >2% nitrogen that displayed good supercapacitor performance and favorable capacitance retention [15]. In spite of the multifaceted application of RTILs in porous carbon tailoring, the pore tuning effect of the water/IL binary mixture is not widely recognized. Yang et al. observed that the added RTIL influenced the pore structure of the developing carbon aerogel already at relatively low concentration (<3 wt % in the initial water-based solution), but this question was not investigated further [13]. 

In this paper, we report a systematic investigation of the role of the RTIL content of the reaction medium on the resulting carbon aerogels over a different and much wider composition range. The series of resorcinol-formaldehyde-based carbon aerogels was synthesized in a water-([emim][EtSO_4_]) binary reaction medium with high RTIL content, which was varied in the range 45–91 wt %. This concentration range allowed us, for the first time, to use as a templating agent not only the ionic liquid but also the supramolecular structure of the binary solvent [16]. 

## 2. Experimental

### 2.1. Sol-Gel Synthesis of the Carbon Aerogels

Resorcinol (R) was dissolved in 3 mL of water-1-ethyl-3-methylimidazolium ethyl sulfate [emim][EtSO_4_] binary solvent, then formaldehyde (F, 37% aq. solution) was added. The concentration of R in the initial solution was 0.14 g/mL, at R/F molar ratio 0.5. No catalyst was used. The initial sol was sealed in glass vials and cured at 85 °C for 7 days. The reusable IL was removed from the wet gels by water extraction. After replacing water by acetone, the latter was removed by supercritical carbon dioxide (scCO_2_) extraction. The dry polymer aerogels were converted to carbon in a rotary quartz reactor under high purity dry nitrogen flow at 800 °C for 1 h. For comparison, a carbon aerogel sample (C100cat) was prepared without IL in the presence of Na_2_CO_3_ catalyst. The resorcinol, formaldehyde, and Na_2_CO_3_ were purchased from Merck (Budapest, Hungary). The ionic liquid and the gases were obtained from Sigma-Aldrich (Budapest, Hungary) and Linde (Budapest, Hungary), respectively. All chemicals used for the synthesis were employed as received. The set of samples prepared is listed in Table 1.

### 2.2. Characterization Methods

The morphology of the CAs was investigated over a wide range of length scales using gas adsorption, scanning electron microscopy (SEM), and small-angle X-ray scattering (SAXS) techniques. Low-temperature (−196 °C) nitrogen adsorption isotherms were measured with a NOVA 2000e automatic analyzer (Quantachrome, Boynton Beach, FL, USA). The apparent surface area *S*_BET_ was calculated using the Brunauer–Emmett–Teller (BET) model. The total pore volume (*V_tot_*) was derived from the amount of nitrogen adsorbed at relative pressure *p*/*p*_0_→1, assuming that the pores were then filled with a liquid adsorbate. The micropore volume (*W*_0_) was derived from the Dubinin–Radushkevich (DR) plot. The volume of the mesopores *V_meso_* was calculated as *V_tot_* − *W*_0,N2_. Pore size distributions were calculated by the Quenched Solid State Density Functional Theory (QSDFT) model. CO_2_ isotherms were measured at 0 °C on an Autosorb-1 instrument (Quantachrome). The transformation of all the primary adsorption data was performed by the Quantachrome software ASiQwin version 3.0. The coefficient of correlation R^2^ of the linear fits (BET and DR) was above 0.995, and the residue of the QSDFT computations was 1%. SEM micrographs were obtained with a field emission SU8030 (Hitachi, Tokyo, Japan) microscope. The SAXS measurements were made in the transfer momentum range 0.08 ≤ *q* ≤ 22 nm^−1^ on the BM02 small-angle scattering beamline at the European Synchrotron Radiation Facility (ESRF), Grenoble. Observations were carried out at 3 sample-detector distances from 162.2 cm to 15.8 cm, and standard corrections were applied for background scattering. Intensities were normalized with respect to a standard sample (lupolen).

Water adsorption/desorption isotherms were measured gravimetrically by equilibrating the carbon aerogel samples in an atmosphere of controlled relative humidity (RH) at 20 °C.

A glassy carbon (GC) rotating disc electrode (RDE, Pine Research Instrumentation, Durham, NC, USA) was used for the electrochemical measurements. Details on the electrode preparation are given elsewhere [17]. The working electrodes were prepared by dispersing 2 mg of powdered carbon in 2 mL mixture of isopropyl alcohol and Nafion® solution. After 30 min sonication, a drop of the suspension was transferred on to the dry mirror-polished GC and dried. The loading was 50 μg/cm^2^. Measurements were made in 0.5 M H_2_SO_4_ electrolyte with a three-electrode system, employing a hydrogen electrode and Pt wire, respectively, as reference and counter electrodes. All potentials are given in the Reversible Hydrogen Electrode (RHE) scale. 

## 3. Results and Discussion

### Morphology of the Carbon Aerogels

The impact of water on the morphology was investigated by changing the composition of the [emim][EtSO4]/water reaction medium over a wide concentration range, a subject that has not hitherto been reported (Table 1). The generally accepted molar ratio 1:2 R/F was, however, employed in the synthesis, and the R content of the reaction mixtures was ca. 20% lower than used elsewhere [13,18]. A further important difference in this work was that after the synthesis, the IL was completely washed out with water prior to supercritical extraction and subsequent pyrolysis. This step was essential in order to determine whether or not the IL was incorporated into the matrix during the synthesis [16]. 

**Figure 1:**
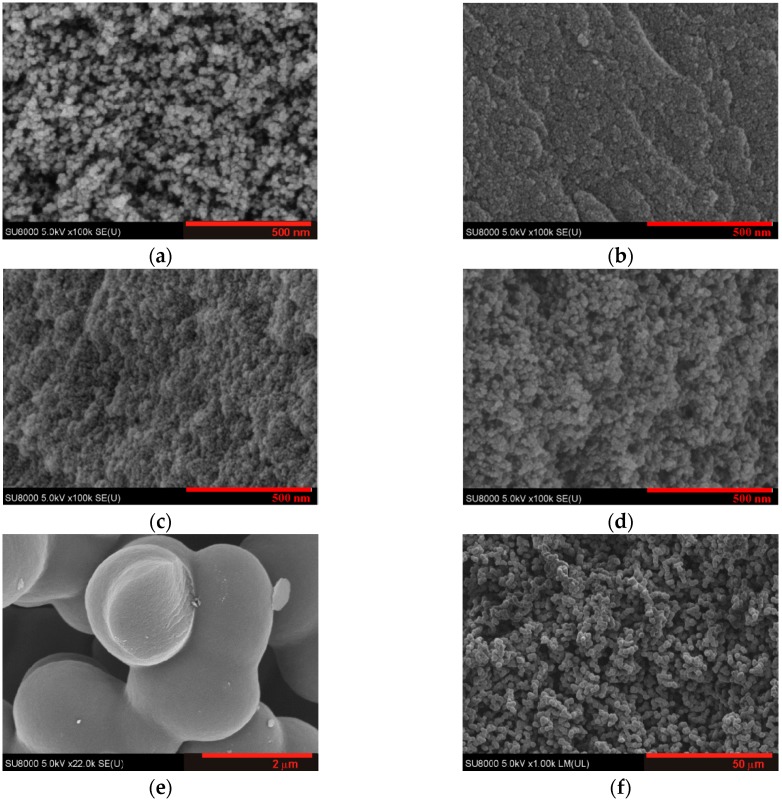
SEM images of selected carbon aerogels. (**a**) C100cat; (**b**) C9; (**c**) C31; (**d**) C43; (**e**,**f**) C55. The scale bar is 500 nm for a–d, 2 μm for e, and 50 μm for f. The average size of the spherical beads is listed in Table 2.

The morphology of the carbon gels was studied on the macro, meso, and nanoscale by imaging, gas adsorption, and small-angle X-ray scattering techniques. Figure 1 shows the 3D structure of the gels, as revealed by scanning electron microscopy (SEM). As the polymerization took place without Na_2_CO_3_ catalyst, it could be concluded that [emim][EtSO4] also acted as a catalyst. The bead size increased with water content, and, at the same time, the structure became less compact. When the water content increased from 43 to 55 wt %, an enormous change was observed, in which the bead size increased by two orders of magnitude (Table 2).

**Figure 2:**
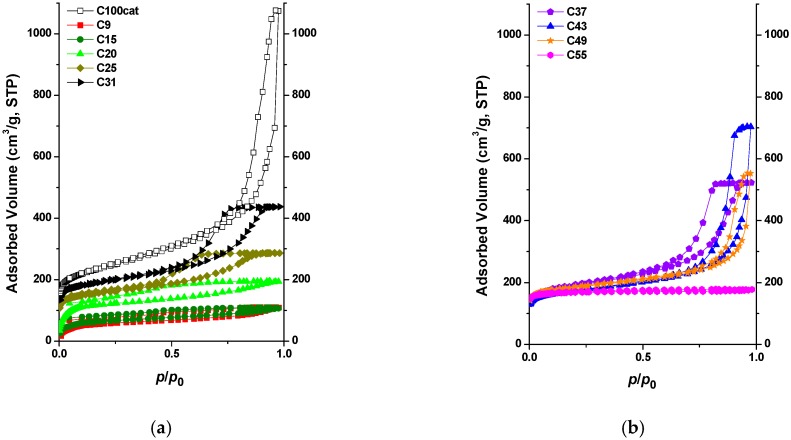
Low-temperature nitrogen adsorption/desorption isotherms of carbon aerogels synthesized in [emim][EtSO_4_]/water medium. For comparison, the isotherm of C100cat is also included. For clarity, isotherms are plotted in two separate diagrams.

**Figure 3:**
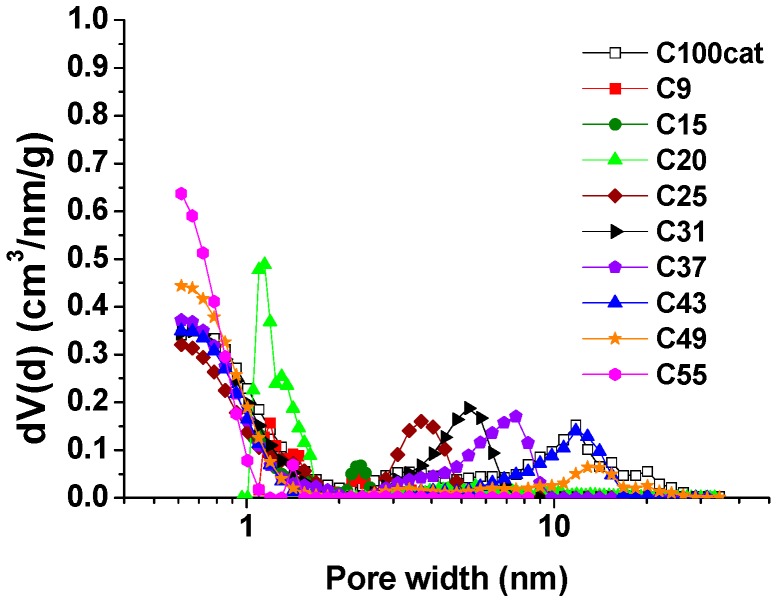
Pore size distribution derived from the nitrogen adsorption data calculated with the Quenched Solid State Density Functional Theory (QSDFT) model (slit geometry).

The changes in structure with increasing water content could also be measured from the shape of the nitrogen adsorption isotherms (Figure 2). The low-pressure hysteresis in the transitional Type I-II isotherms of samples containing up to 20% water content is the signature of poorly developed pore structures [19]. Further increase in the water content of the synthesis medium led to an isotherm of Type IVa, which is evidence of the microporous-mesoporous nature of the carbons, in corroboration of the macropores observed in the SEM images. Interestingly, the micropore region of the nitrogen isotherms remained constant. This included the ultra-micropores, which added ca. 20% to the pore volume of the well-developed samples. The total pore volume, by contrast, and, therefore, the volume of the mesopores, increased continuously up to 43% water, after which it dropped abruptly and disappeared completely: the carbon obtained with 55% water contained no mesopores (Figure 3). The shape of the hysteresis loops indicate an interconnected pore network. The gradual shift of the loop position towards higher relative pressures and its change in shape from Type H2a to H1 with more parallel vertical branches suggest that the mesopores become wider, but with a narrower distribution. In this case, the Type H1 hysteresis suggests a network of ink-bottle pores with a neck size distribution similar to that of the width distribution [19].

This means that the pore morphology of these carbon aerogels, i.e., the volume and width distribution of the pores, can be sensitively tailored by the water content of the reaction medium. Once a stabilized pore structure is obtained, the apparent surface area remains practically constant. Liquid exchange of water by acetone, followed by supercritical removal of the latter, finally led to a porous carbon of surface area and porosity similar to those of the post-treated xerogels [14,18]. 

**Figure 4:**
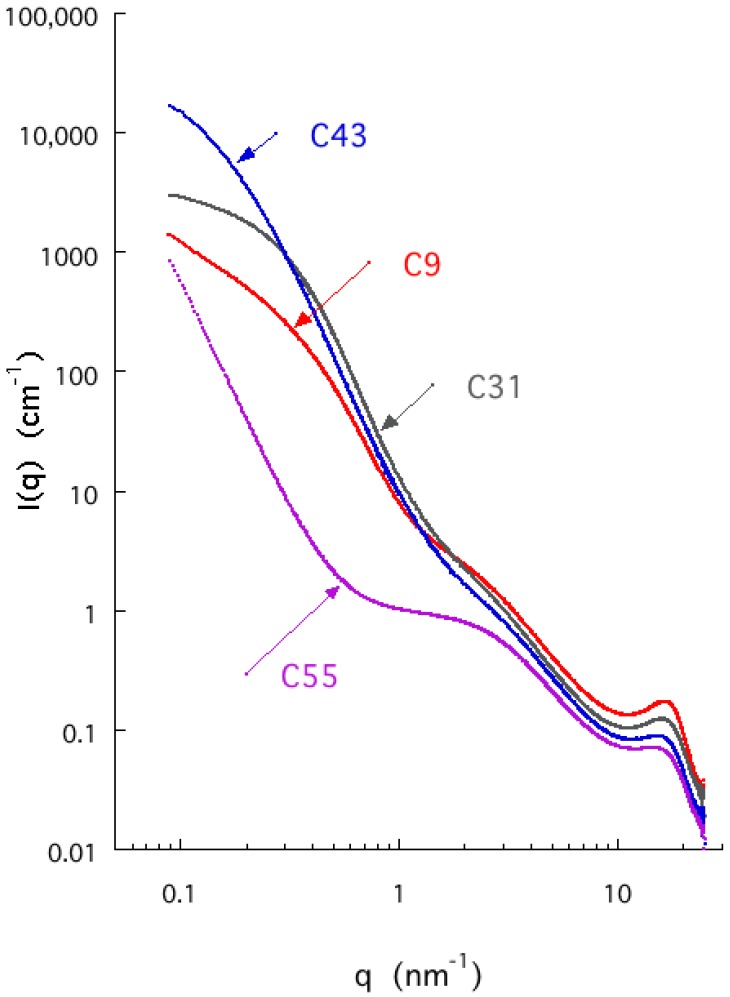
SAXS response of selected carbon aerogels prepared in various [emim][EtSO_4_]/water media.

The SAXS response curves measured in the selected samples are shown in Figure 4. They all share the same general features, in which the broad peak in the high *q* region (*q* ≈ 17 nm^−1^), characteristic of amorphous carbon, is an analog of the 0.335 nm interlayer spacing in crystalline graphite. Apart from sample C55, all curves display two shoulders in the lower *q* region. The intermediate *q* shoulder defines the porous nature of the carbons, while the curvature of the shoulder at lowest *q* indicates the presence of bead structures having a radius of gyration *R_G_* ≈ 10 nm, in qualitative agreement with the estimated diameter *d_SEM_* found by scanning electron microscopy (Table 2). The steep power-law behavior of slope −4 in the scattering curve of C55 at low *q* is characteristic of surface scattering from very large structures. The absence of either a shoulder or even deviation from the power-law indicates that the size of these structures is much greater than the largest size window 1/*q_min_* explored in the present SAXS measurements. This finding is in agreement with the gas adsorption measurements. 

The surface area

*S_x_* = (π*K*/*Q*) [*V_tot_*/(1 + *V_tot_* ρ_He_)] (1)

listed in Table 2, is calculated from these curves, as described previously [20]. In Equation (1), the Porod final slope *K* is measured in the microporous region *q* ≤ 1 Å^−1^. The total integrated intensity

(2) $$Q=\int_{0}^{\infty }\left(I\left(q\right)-b\right)q^{2}dq$$

is found by extrapolating the SAXS curves to *q* = 0, where we assume that the intensity *I*(*q*) is independent of *q* below *q* ≤ 10^−3^ nm^−1^. The quantity *b* is the incoherent signal from atomic disorder in the amorphous structure and is found from the high *q* region where the intensity varies as [21]

*I*(*q*) = *Kq*^−4^ + *b* (3)

Measurement of the helium density in one member of this set of samples by gas pycnometry yielded ρ_He_ = 2.13 g/cm^3^. Since the quantity of material was, however, insufficient to perform pycnometric measurements in each case, this value was used throughout.

The discrepancy between the surface areas obtained from adsorption and X-ray scattering indicates that samples C31 and C43 and, especially, C9 possess a substantial fraction of closed or inaccessible pores [20]. This observation implies that the internal structure of the beads contain more defects when the water content in the precursor sol is low, as corroborated by the low-pressure hysteresis in these samples. SEM imaging, adsorption, and X-ray scattering provides information about the samples at different length scales. According to Figure 1, the size of the spherical beads increases with increasing water content. The beads themselves become more compact, as revealed by the ratio of the surface areas derived from SAXS and adsorption, respectively. It decreases gradually from ca 5 to ca 1. Conversely, the space between the beads increases, resulting in an exceptionally supermacroporous structure in sample C55. 

**Figure 5:**
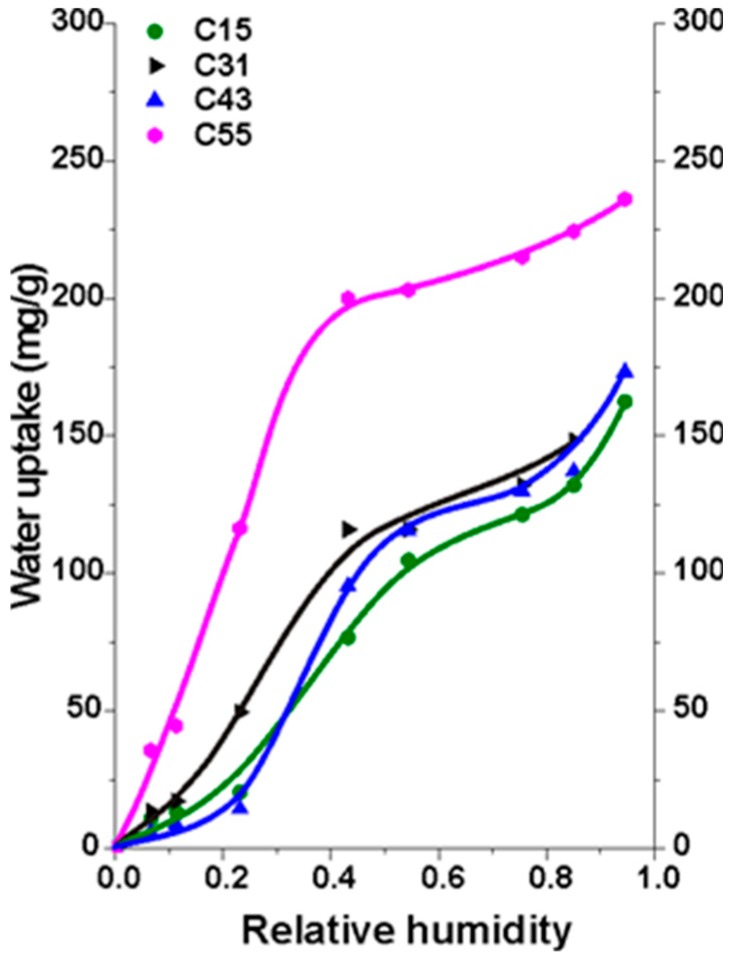
Water vapor isotherms of selected carbon aerogels at 20 °C.

Gravimetric water vapor adsorption isotherms were measured on selected samples. Removal of the ionic liquid prior to the pyrolysis provides information on its reactivity during the polycondensation reaction. As the nitrogen and sulfur content—if any—of the samples was below the detection limit of the XPS method, we conclude that the ionic liquid does not participate in the polymerization and is completely removed during the solvent exchange steps prior to carbonization. It is, therefore, reasonable to conclude that the differences between the adsorption isotherms are attributable to the pore structure. Figure 5 implies that water uptake in the supermacroporous C55 sample is not hindered by diffusion between the narrow and mainly hydrophobic pore walls.

**Figure 6:**
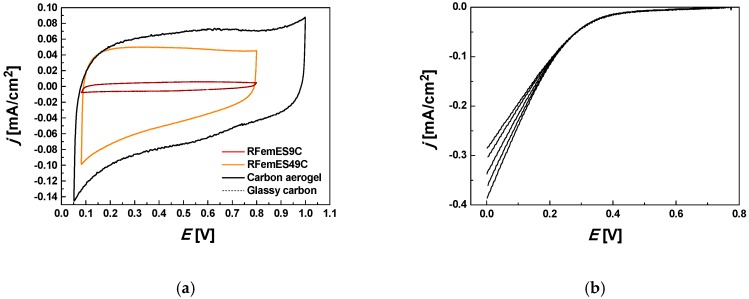
Cyclic voltammograms of samples C9 and C49 measured in Ar-purged 0.5 M H_2_SO_4_ at scan rate 50 mV/s (**a**). Carbon aerogel from [17] is plotted for comparison. (**b**) Linear sweep voltammograms of C49 measured in O_2_ saturated 0.5 M H_2_SO_4_ at scan rate 10 mV/s.

The electrochemical characteristics of the exclusively microporous C9 sample having the smallest measurable surface area and the mesoporous C49 sample with the largest surface area were investigated (see Figure 6). The powdered electrode materials were tested in a three-electrode cell configuration. The corresponding cyclic voltammograms (CV) are displayed in Figure 6a. The measurements were recorded at 50 mV/s in Ar-purged 0.5 M H_2_SO_4_. The curve of the C9 sample is practically identical to that of the glassy carbon holder, i.e., the electrochemical surface area of the sample is very small. This finding implies that the electrolyte is unable to penetrate the pores, either because they are very narrow or because of the high proportion of closed pores, thus maintaining contact only with the geometrical surface of the powdered carbon. The effect could be related to structural defects in C9, as suggested by the anomalously high value of S_SAXS_/S_BET_. In the case of the mesoporous sample C49, the curve has a distorted rectangular shape, reflecting the contribution of the pseudo-Faradaic reactions of the surface functions [22]. Owing to the greater accessible surface of this sample, the CV hysteresis is much wider. This response is also comparable to that of the conventional resorcinol-formaldehyde-based carbon aerogels synthesized in water and has a similar value of *S*_BET_ (≈800 m^2^/g) [17,23]. 

Since the cathodic hump (see Figure 6a) could not be ignored, linear sweep voltammograms were measured on a rotating disc electrode (RDE) in O_2_-saturated 0.5 M H_2_SO_4_ electrolyte. The polarization curves of C43 at different potentials and rates of rotation (225–1225 rpm) are shown in Figure 6b. At low potentials, the current densities depend on the rate of rotation, indicating that the oxygen reduction is diffusion-limited. The electrochemical performance of these carbon aerogels is inferior to that of the carbons reported in the above-cited reference works, principally because of the absence of heteroatoms other than oxygen. As already noted, in those syntheses where IL was used, Na_2_CO_3_ catalysts were also employed, and none was removed prior to the pyrolysis [11,13,15]. In several cases, metal contamination (Fe, Ni, Co) was introduced intentionally in order to enhance oxygen reduction activity [10,14]. 

## 4. Conclusions

Observations of resorcinol-formaldehyde-based carbon aerogels obtained by the carbonization of the polymer aerogels synthesized in water-ionic liquid binary mixtures with no added catalyst confirm that the pore size distribution depends critically on the water content. This means that the supramolecular structure of the mixed solvent can be used as a fundamental texture formation tool. The size and volume of the mesopores are tuned easily by changing the composition of the solvent. The carbon aerogels prepared at low water content (up to 20 wt %) exhibit low-pressure hysteresis, while the pore structure becomes consolidated only at higher initial water content. At the lowest water content (9 wt %), the comparison between the internal surface area measured by SAXS and N_2_ by adsorption indicate a substantially higher proportion of closed pores. An abrupt change in the morphology and the surface chemistry is observed at 55 wt % water content. In this sample, practically no mesopores were detected, and the water vapor adsorption isotherm shows it to be hydrophilic. For electrochemical applications, however, the observed performance indicates a need for additional treatment, and that structural defects can compromise the performance.

## Figures and Tables

**Table 1:** List of prepared carbon aerogel samples.

Sample	Initial Water Content of the Solvent (wt %)
C9 *	9.1
C15	15.4
C20	19.7
C25	25.2
C31	30.9
C37	36.7
C43	42.6
C49	48.7
C55	55
C100cat	100 **

* added with the aq. formaldehyde; ** with Na_2_CO_3_ catalyst.

**Table 2:** Characterization of morphology. Data from SEM, gas adsorption, and SAXS analysis *.

Sample	*d*_SEM_^a^	*S*_BET_^b^	*V*_tot_^c^	*W*_0,N2_^d^	*V*_meso_^e^	*W*_0,CO2_^f^	*S*_SAXS_^g^	*R_G_*_,SAXS_^h^	*S_SAXS_* *S_BET_*
	nm	m^2^/g	cm^3^/g	cm^3^/g	cm^3^/g	cm^3^/g	m^2^/g	nm	
C9	12 ± 2	199	0.17	0.08	0.09		1064 ± 50	8.7	5.25
C15	–	267	0.16	0.09	0.07	0.063	–	–	
C20	–	469	0.30	0.20	0.10	0.053	–	–	
C25	–	590	0.44	0.23	0.21	0.063	–	–	
C31	14 ± 2	713	0.68	0.28	0.40		1417 ± 30	7.3	1.99
C37	–	697	0.81	0.27	0.54	0.069	–	–	
C43	20 ± 3	644	1.1	0.25	0.85	–	1494 ± 30	16.8	2.32
C49	–	766	0.85	0.27	0.58	0.079	–	–	
C55	1977 ± 238	677	0.27	0.26	0.10	0.089	787 ± 80	n.a. ^j^	1.16
C100cat	20 ± 4	865	1.7	0.35	1.35				

* SEM: scanning electron microscopy; SAXS: small angle X-ray scattering_;_^a^ diameter of the spherical beads from SEM (from 100 data); ^b^ apparent surface area from BET model, the estimated error is 5%; ^c^ total pore volume from N_2_ adsorption; ^d^ micropore volume from N_2_ adsorption; ^e^ mesopore volume, *V*_meso_ (= *V*_tot_ − *V*_micro_); ^f^ ultramicropore volume from CO_2_ adsorption; ^g,h^ surface area and radius of gyration of the elementary building units from SAXS; ^j^ out of the range of the measurement. Note that since *R_G_*_SAXS_ is estimated from the region of maximum curvature in the low *q* SAXS response (Figure 4) (i.e., where *qR*_G_ > 1), the error in the values can be as large as 20%. The estimated error in *S*_SAXS_, which stems principally from the extrapolation to *q* = 0 in the calculation of the Porod invariant *Q* (Equation (2)), is of the order of 5%, except in sample C55, where the uncertainty could be as high as ca. 10%.
